# Power quality approximation for household equipment load combinations using a stepwise growth in input parameters of AI models

**DOI:** 10.1038/s41598-022-21812-1

**Published:** 2022-11-08

**Authors:** Ladislav Zjavka

**Affiliations:** Department of Computer Science, Faculty of Electrical Engineering and Computer Science, VŠB-Technical University of Ostrava, 17. listopadu 15/2172, Ostrava, Czech Republic

**Keywords:** Energy science and technology, Mathematics and computing

## Abstract

Detached off-grids, subject to the generated renewable energy (RE), need to balance and compensate the unstable power supply dependent on local source potential. Power quality (PQ) is a set of EU standards that state acceptable deviations in the parameters of electrical power systems to guarantee their operability without dropout. Optimization of the estimated PQ parameters in a day-horizon is essential in the operational planning of autonomous smart grids, which accommodate the norms for the specific equipment and user demands to avoid malfunctions. PQ data for all system states are not available for dozens of connected / switched on household appliances, defined by their binary load series only, as the number of combinations grows exponentially. The load characteristics and eventual RE contingent supply can result in system instability and unacceptable PQ events. Models, evolved by Artificial Intelligence (AI) methods using self-optimization algorithms, can estimate unknown cases and states in autonomous systems contingent on self-supply of RE power related to chaotic and intermitted local weather sources. A new multilevel extension procedure designed to incrementally improve the applicability and adaptability to training data. The initial AI model starts with binary load series only, which are insufficient to represent complex data patterns. The input vector is progressively extended with correlated PQ parameters at the next estimation level to better represent the active demand of the power consumer. Historical data sets comprise training samples for all PQ parameters, but only the load sequences of the switch-on appliances are available in the next estimation states. The most valuable PQ parameters are selected and estimated in the previous algorithm stages to be used as supplementary series in the next more precise computing. More complex models, using the previous PQ-data approximates, are formed at the secondary processing levels to estimate the target PQ-output in better quality. The new added input parameters allow us to evolve a more convenient model form. The proposed multilevel refinement algorithm can be generally applied in modelling of unknown sequence states of dynamical systems, initially described by binary series or other insufficient limited-data variables, which are inadequate in a problem representation. Most AI computing techniques can adapt this strategy to improve their adaptive learning and model performance.

## Introduction

Autonomous off-grids need to optimize the energy supply with load scheduling in a 24-h plan, i.e. forecast and balance their daily RE generation and consumption. Fully self-producing power units must control the PQ in a mid-time horizon considering the optimal utilization of RE sources to minimize unwanted events and signal spikes in the electrical components. Detached smart houses must stabilize their energy production by using a supporting accumulator storage to compensate for uncertainty and fluctuations in RE power depending on local potential. Stable energy supply determines the functionality of electrical equipment and appliances according to PQ monitoring much more than in conventional grids. Novel AI techniques can be used in the estimation of PQ to optimize the parameters in scheduling active load appliances in the next switching-time states, ensuring PQ acceptable ranges for off-grid systems whose behavior is impossible to define exactly. Complete system states are unknown for varying environments and operating plans, determined by the external RE supply limitations and characteristics of the applied system components. Future states of autonomous systems must be estimated and resolved in unknown cases, which is unusual in standard grid networks. Optimal consumer load (re)scheduling according to the peak RE power supply, using a time-shifting plan, can be practiced in real operation modes using the estimated PQ and RE data. Real-time load planning or PQ monitoring, considering predicted RE supply and backup state storage, is necessary to compensate for power production instabilities in user demands and secure failure-free operation of autonomous grids^1^.

The characteristics of household appliances must be evaluated in the preparation of load plans for the RE potential and various (re)charge states^2^. Specific load appliances in various sequenced combinations of switching time modes affect the PQ and subsequent system states^3^. Automatic detection of PQ events and anomalies with full self-control in user-adaptable power demand scenarios is also important from the point of integration of distributed RE supply in complex grids^4^. Active management of power demand enables efficient detection and reduction of undesirable PQ events in load planning and RE utilization^5^. Several publications only attempt to estimate PQ variances and irregularities on the daily or midterm horizon, though their solutions are inevitable in operation and early warning in smart systems. Detection of PQ distortion waves is fundamental in the regulation of RE power consumption and optimization of load scenarios^6^. A unit based on 2-D dynamic programming can recognize an acceptable variation range of power demand and battery state of charge in a longer-term horizon, to secure efficient operation of each module.

Neuro-Fuzzy controllers can determine the operation modes of components in microgrids in a shorter time, by evaluating the real conditions and constraints of the system^7^. Smart meters allow demand operators to apply PQ indices in residential house distribution networks, based on the computing of probabilistic intervals for models with harmonic data distortion^8^. Remote microgrids can be represented by clustering techniques, considering the distribution distance and source potential, to be supplied with additional information to improve stability and efficiency in energy utilization and shared power exchange. Hierarchical strategies based on model decentralization provide distributed control at smart levels with power backup and exchange in units through efficient energy management^9^. The measurement information approach in modeling optimizes and corrects the energy consumption plan in building networks. The voltage index can determine the overall energy efficiency in smart buildings in the analysis and quantification of PQ signals^10^. Two key types of irregularities can be identified in analyzing the solar production capacity of low-power PV grids: certain and uncertain events; considering deterministic, statistics, or probabilistic signal processing^11^.

**Figure 1:**
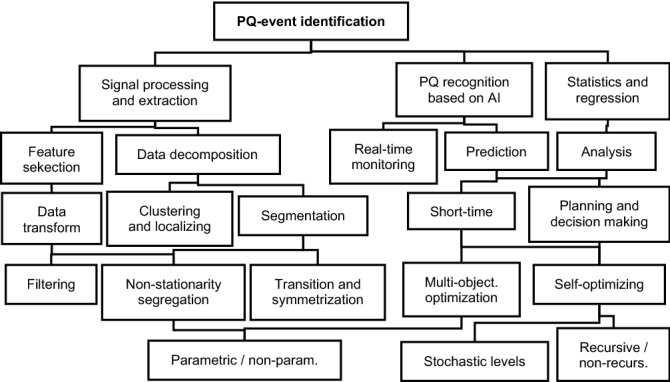
Application of pre-processing and AI modelling techniques in PQ-event detection, divided in consideration of their characteristics and particular usability criteria.

Optimization, identification, and real-time monitoring of PQ can be done by hybrid AI algorithms usually fusing the two basic groups of data preprocessing and machine learning methods to detect/predict the sources of PQ events which are classified into stationary/nonstationary irregularities in general (Fig. 1). The phase angle can be used to relate PQ disturbances in different faults of asymmetric energy events. The localization time index results from the Hilbert/Stockwell transformations applied to PQ signals. The disturbances are recognized and localized taking into account the calculated index maxima^12^. Clustering techniques are usually used to partition PQ data into main groups in cases if information (of the desired output) is available. Hybrid AI approaches fuse various data pre-processing and optimization techniques to identify eventual PQ anomalies^13^. Finite element control can eliminate wave distortion and noise in harmonic signals to be further applied in conditioned prediction^14^. Photovoltaic (PV) panels unbalance the harmonics of PQ signals by increasing the temporal variability. Probabilistic intervals can be analyzed to re-assess the uncertainty in PV power supply at different levels of RE production and load schemes^15^. Neighbor adaptive sorting recognizes block and merge amplitudes in signals to locate potential sources of PQ disturbances. Data-driven modeling can transform and segregate signals into categorical levels to identify errors related to environment conditions^16^. Downsampling, based on empirical mode decomposition, can be used to calculate the characteristics of the data to better detect PQ-distorted signals^17^. A visual attention technique can select data feature indices, in the fusion of PQ detection and scale analysis, using processed images of the original signals^18^. Adaptive resolution based on the S transform can be used to analyze the PQ data in the frequency-time domain. The multi-objective window optimizer improves the performance of PQ models in relation to the maximum concentration energy^19^. Principal component analysis (PCA), applied together with K-means clustering, can be integrated into a search algorithm for atypical patterns in PQ variances in short times^20^. Statistical distribution characteristics of data can be computed according to sparse-envelope Kurtosis index maxima. The extracted features are then applied in a kernel classifier that uses a functional link network with random vectors^21^. Atomic decomposition algorithms refine PQ data to produce physically clear signals. The convolutional neural network model can then be used to select subspaces in each atomic predictor to reduce the search for computational and optimization time^22^. The combination sequence of power load appliances, active in the off-grid, can be defined as binary data. No additional PQ values are available for the next grid states in load planning to be processed in the prediction. However, the PQ-training data can be measured without problem, but the unseen load scheme input series do not comprise any. The learning process with only input of binary codes ‘1/0’ is generally inefficient in modelling real-world data and does not allow adequate pattern representation for unknown load sequenced combinations (applied as input to the model in the new estimates). The proposed multistage procedure selects and increases progressively PQ inputs, used to develop more complex structural models in training, able to approximate the unknown load PQ data in better detail. Approximate PQ data for selected input parameters, correlated with the load codes, are calculated before the consequent processing in the next estimate stage to obtain better quality in the model target output. This approach enables us to train PQ-data samples, added to the input vector, including initial load codes, to process the approximate PQ series as model input in the next load estimates. This quality improvement algorithm produces more capable models in each subsequent iteration step to obtain a better approximation of the PQ data, determined by the unknown load series in detached systems. It is applicable to improve solutions of the over-viewed publication methods. Multistep PQ prediction was performed and tested on data sets recorded in an experimental off-grid without producing RE, as PV panels were disconnected, so weather conditions are irrelevant in this study. Several types of single-model recent AI machine learning and statistics methods using no data preprocessing, e.g. transformation, decomposition, etc., were compared and evaluated in the modelling of PQ parameters. Overall data measurements for all possible combination load sequences allow adequate training and testing in the proposed scheme.

The designed procedure improves step by step the accuracy of AI self-optimizing models evolved in several hierarchical levels. Its key characteristics are listed below.

- Starting with the low-level models, applying the input binary data only, to code active load components, as PQ-data are unknown in the next prediction state of a schedule plan.
- Binary codes of active load consumers, inadequate in the representation of real data patterns, define the model input in the first stage to be extended with PQ approximates.
- The progressively expanded model input vector (with selected PQ-parameters) enables to evolve more advanced model structures in each iteration to better estimate the target PQ output.
- A more complex representation of the problem allows us to train data samples of the added PQ inputs and subsequent processing of the PQ replacement series to compute the target output.
- The step-by-step expanded AI model vector enables the use of approximate support data, where the real-world series are unknown in the next states.

## Data measurement and PQ-standards in smart grids

**Figure 2:**
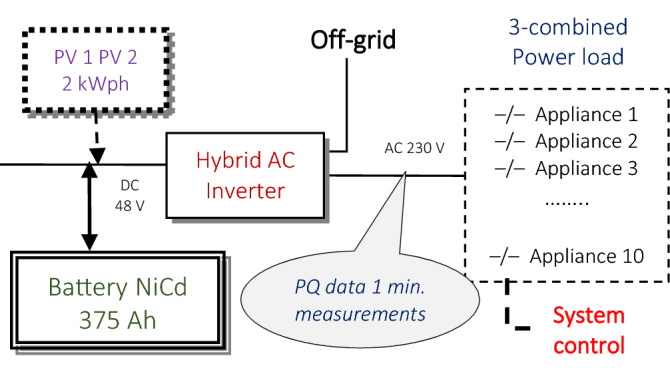
The off-grid with 10 power consumers (PV panels are not applied).

The detached off-grid system has two connected PV panels, each of which can induce 2 kWph in peak power. Photovoltaic power was not supplied at the time of measurement experiments to ensure the same condition (100$ fully recharged battery) in modelling deterministic states for all the simulated load combination sequences. The power load of three combined turn-on electrical appliances of 10 types in all, including: mower, drill, heating, TV, fridge, lighting, kettle, boiler, microwave, switching; is supported from the backup Ni–Cd accumulator bank (Fig. 2). The voltage, current, frequency, and phase data were measured on the hybrid AC converter to recognize the real and apparent power. The PQ parameters for all possible 120 variations of the 3-load sequences were saved in the database in a time of 12 min for 1 min stamped series. After 12 min. measurement time for every 3-combination load of binary coded power consumer, the battery was 100% fully recharged for a capacity of 375 Ah in the remaining 18 min. time to finish the 30 min. cycle and obtain the same system condition for the following load PQ measurements. Restoring the same state of charge (SoC) of the power system to record the PQ series for each 3-consumer load binary sequence reduces the intermitted character of the RE supply [H].

The experimental off-grid system (Fig. 2) uses:

- AC hybrid inverter with the battery bank
- PQ measuring devices

The hybrid inverter is of type Conext XW + 8545 with a continuous output power of 6.8 kW. It was used to control the charging of the battery bank comprising 37 Ferrak 375 KPL batteries, where each NiCd battery had a capacity of 375 Ah and a nominal voltage. Each cell of the 100% charged battery can produce about 1.5 V, while the fully discharged voltage state is close to 1 V. These data are valid only if the discharge current value is close to 0.1C. Therefore, the experiments were carried out on 100% SoC to avoid a low-output current from the AC hybrid inverter. The equipment and AC distribution modules were connected to the hybrid inverter through a switchboard control system that identified the ignition of each 3 combination loads of the appliances in the defined charging schedule. The PQ data measurements were automatically recorded in a database system. The PQ measurement was performed with the KMB SMC 144 device on a one-minute time scale. Minimum, maximum and average values of the PQ parameters were calculated and saved for each minute in a data vector^3^.

The 2 PVP panels were not connected to produce energy for the power supply. Voltage (U), Power Factor (PF), Power (P), Frequency (Freq), Short-Term Flicker Perceptibility (Pst) and THDU (Total Harmonic Distortion of Voltage) data were measured and averaged in 1 min series in the off-grid for all attached experimental equipment load to be automatically stored by a control software in the database. The PQ parameters were after-estimated by AI models for various unlearned 12-min. switching-time power load of 3-attached electrical appliances, not considering the recharge time in 18 min, to simulate real operational conditions in a smart microgrid [H].

The PQ standards define the power characteristics to avoid system failures, dropout, and loss in performance. The European standards: EN 50160 and EN 61000 normalize the technical conditions of PQ in power systems, equipment compatibility, and measurement protocols. Classification and detection of PQ events is possible in relation to the data quantity thresholds concerning the system states and nominal time. PQ abnormalities in nominal states can corrupt or limit the desired life of electrical equipment and appliances. They represent unacceptable very short-time fluctuations or ramp-spikes in PQ-parameters in comparison with the standard wave signals. The abnormal magnitudes are largely induced by switching components in a sequenced load, which is obvious in detached off-grid systems dependent on RE. Incompatible control modules can produce variations in harmonics and flickers with unbalanced load and frequency regulation^6^. The most important PQ parameters are calculated from the relative amplitude and fundamental frequency waves^23^. Converters in energy transmission and distribution with non-linear loads can induce sag, swell, impulsive, or transient oscillatory harmonics, and signal interruption^24^.

## AI methods used in equipment load PQ prediction

### Differential learning a novel neuro-computing strategy

**Figure 3:**
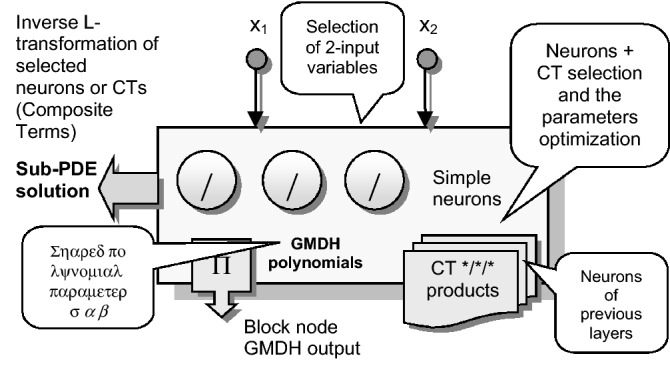
Node blocks form and convert specific 2-variable sub-PDEs.

Differential learning employs self-organization in node-by-node expanded synchronized structure of Polynomial Neural Network (PNN) in evolvement modular components. Each PNN selected module can be added to the output sum (Fig. 3), solving the particular 2-variable Partial Differential Equations (PDEs) defined in the node.

Node-defined sub-PDEs are polynomial-converted into the Laplace form using the adapted functional derivative transformation of Operator Calculus (OC) (1).

1 $$L\left\{ {\left. {f^{(n)} (t)} \right\}} \right. = p^{n} F(p) - \sum\limits_{k = 1}^{n} {p^{n - i} f_{0 + }^{(i - 1)} } \begin{array}{*{20}c} {} & {} \\ \end{array} L\left\{ {\left. {f(t)} \right\}} \right. = F(p)$$

*f*(*t*), *f*’(*t*), …, *f*^(*n*)^(*t*) are continuous originals in the interval <0+ *,* ∝> *, p and t* are complex and real valued variables.

The inverse L-transform (2) is applied to the reduced-ratio images to restore the originals of unknown functions, whose sum gives the searched separable node series.

2 $$F(p) = \frac{P(p)}{{Q(p)}} = \sum\limits_{k = 1}^{n} {\frac{{P(\alpha_{k} )}}{{Q_{k} (\alpha_{k} )}}} \frac{1}{{p - \alpha_{k} }}\begin{array}{*{20}c} {} & {} & {} \\ \end{array} f(t) = \sum\limits_{k = 1}^{n} {\frac{{P(\alpha_{k} )}}{{Q_{k} (\alpha_{k} )}}} e^{{\alpha_{k} \cdot t}}$$

where P(p), Q(p) are multinomials of degree s-1, s.

Complex Neural Networks (CNN) apply the Euler data representation which can be used in solving node sub-PDEs. Differential Polynomial Neural Network (D-PNN) partitions the n variable PDE of the general k-th order of (3) into a separable series (4) of 2-variable PDEs (5), defined at the nodes, to solve them using the above OC procedure^25^.

3 $$a + bu + \sum\limits_{i = 1}^{n} {c_{i} \frac{\partial u}{{\partial x_{i} }}} + \sum\limits_{i = 1}^{n} {\sum\limits_{j = 1}^{n} {d_{ij} \frac{{\partial^{2} u}}{{\partial x_{i} \partial x_{j} }}} } + \cdots = 0$$

4 $$u = \sum\limits_{k = 1}^{\infty } {u_{k} }$$

*u(x)* = *f(x*_*1*_*, x*_*2*_*, …, x*_*n*_*)* is the searched *n*-variable separable function*; a, b, c(c*_*1*_*, c*_*2*_*,, …, c*_*n*_*), d(d*_*11*_*, d*_*12,*_* …), …* are coefficients and *u*_*k*_ are sum series of *u* function.

5 $$A(\partial^{2}u/\partial x^{2}1)+B(\partial^{2}u/\partial x1x2)+C(\partial^{2}u/\partial x^{2}2)+D(\partial u/\partial x1)+E(\partial u/\partial x2)+Fu+G=0$$

A, B, C, D, E, F, G are functions of u, x_1_, x_2_—inputs.

This procedure can be efficiently used in modelling complex and high-dynamic unknown systems. PNN performs feature extraction from dozens of variables. Each added node searches for the optimal 2-inputs to solve a pre-defined sub-PDE in the progressively expanded PDE modular model. The selected node components (6), i.e. derivative neurons, can be included in the sum model and adapted in each training iteration to minimize calculated errors^26^.

6 $$y_{1} = w_{1} \frac{{b_{0} + b_{1} x_{1} + b_{2} sig(x_{1}^{2} ) + b_{3} x_{2} + b_{4} sig(x_{2}^{2} )}}{{a_{0} + a_{1} x_{1} + a_{2} x_{2} + a_{3} x_{1} x_{2} + a_{4} sig(x_{1}^{2} ) + a_{5} sig(x_{2}^{2} )}} \cdot e^{\phi }$$

*y*_*i*_ are particular sub-PDE solutions of node searched functions*, arctg(x*_*2*_*/x*_*1*_*)* is the *ϕ* phase of 2-inputs *x*_*1*_*, x*_*2*_*; sig()* is a sigmoidal transform of square variables; *a*_*i*_*, b*_*i,*_* and w* – member parameters and term weights.

Gradual modular extension and error minimization can lead, according to Goeddel’s theorem of incompleteness, to optimal pattern model in adaptation to input–output data samples. As D-PNN applies the L transformation to PDE derivatives and a self-selection of 2-input nodes, no signal pre-processing or feature extraction is needed^27^. D-PNN gradually forms a binary PNN tree structure. New nodes are added in the following layers to expand the synchronized sum series model with the selected PDE modules and obtain a better approximation of the desired output^28^.

**Figure 4:**
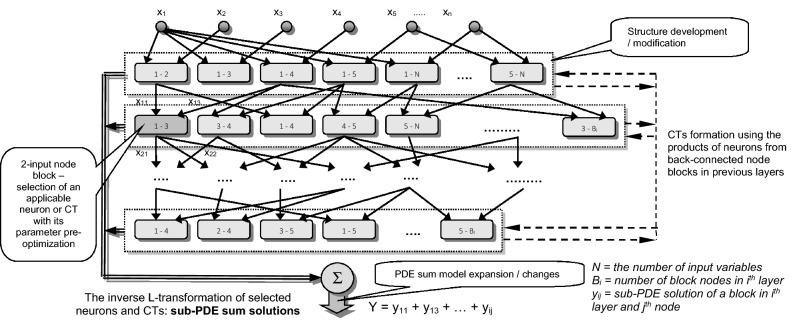
D-PNN searches for the optimal 2-input node in each layer to produce neurons or CTs in the blocks to be included in the output sum.

**Figure 5:**
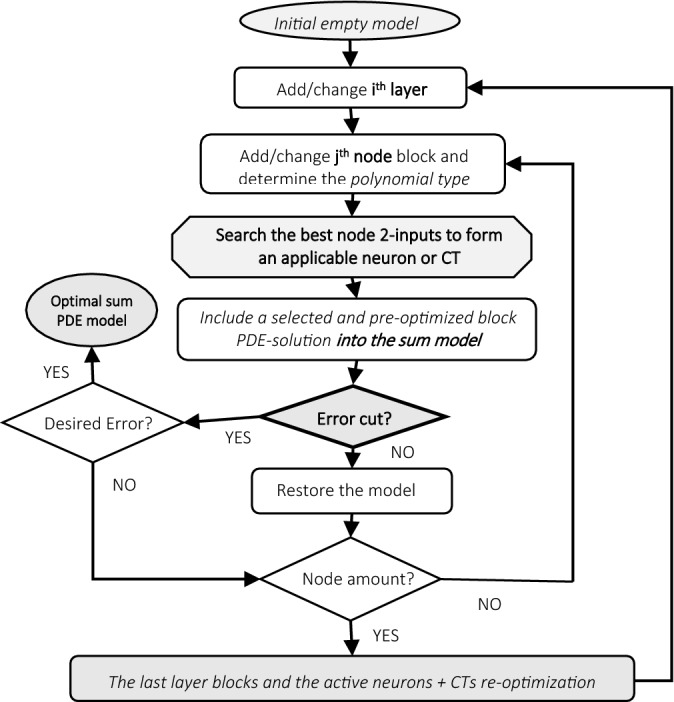
Development of D-PNN gradual models using new added sum PDE components formed in nodes of the parallel expanded PNN structure.

Blocks group possible PDE-solutions in the nodes to form simple neurons or composite terms (CTs), formed in relation to variables of the previous layer. The best PDE-component is inserted into the network sum output. Active block nodes are processed in the iterative optimization process to select a neuron or CT that best contributes to the D-PNN output and can be included or removed from / from the model (Fig. 4). Optimal first- or second-order polynomials are composed, and their members are selected to best solve particular 2-variable PDEs at the nodes. A node iteration adaptation algorithm skips sequentially or randomly the blocks of neurons and CTs in the D-PNN tree structure to minimize the error in training coordinated with the testing based on external complement (Fig. 5). This approach determines the update of parameter terms that minimizes training and testing inaccuracies^29^.

Features of the neuro-inspired designed D-PNN. It:

- forms node-by-node the optimal tree structure to parse the n-variable PDE into low-order sub-PDEs of 2-inputs using the adapted OC procedure.
- produces sub-PDE modules from selected basic rational, power, or periodic OC substitution functions in selected tree PNN nodes.
- Expands step-by-step the initial ‘zero’ PDE model using Goedel's theorem, progressively increasing its complexity in minimizing the defined criteria.
- uses a new designed optimization framework that inserts the most valuable PDE components by removing redundant ones from previous layers to synchronize their active application, reducing modular model complexity.

### Statistics and machine-learning regression of Matlab

**Figure 6:**
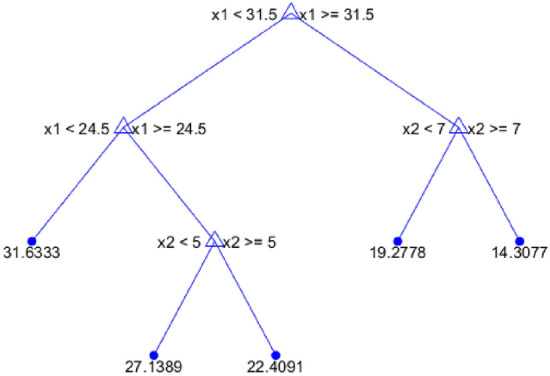
An example of the binary-tree structural model.

The Matlab Statistics and Machine Learning Toolbox (SMLT) for regression involves AI soft computing AI and standard mathematical methods. The best operating models were recognized by considering the minimal test error criteria at each prediction PQ level [A]. SMLT includes:

- **Binary tree—**The optimal branches are followed in the tree structure from the root to leaf nodes that define the output variable. The predictor values are checked in each node to select which one of the 2 branches to follow. The final response is assigned by the value of an end leaf node (see, e.g., Fig. 6).
- **Support Vector Machine (SVM)—**Support vectors are data points placed outside the output interval determined by the ε-parameter for all training points x as flat as possible. Kernel functions determine the nonlinear transformation applied to input data prior to SVM learning [B].
- **Gaussian Process Regression (GPR)**—The model response is calculated as a probability distribution function over the definition space. The GPR basis defines the exact form of a prior mean function. The kernel functions determine the input correlations in the output calculation as a function of distance [D].
- **Ensemble Boosted or Bagged Tree (EBT)—**EBT ensemble models combine results from a set of single weak tree-learners by applying least-squares boosting, bootstrap aggregating or bagging.

**Figure 7:**
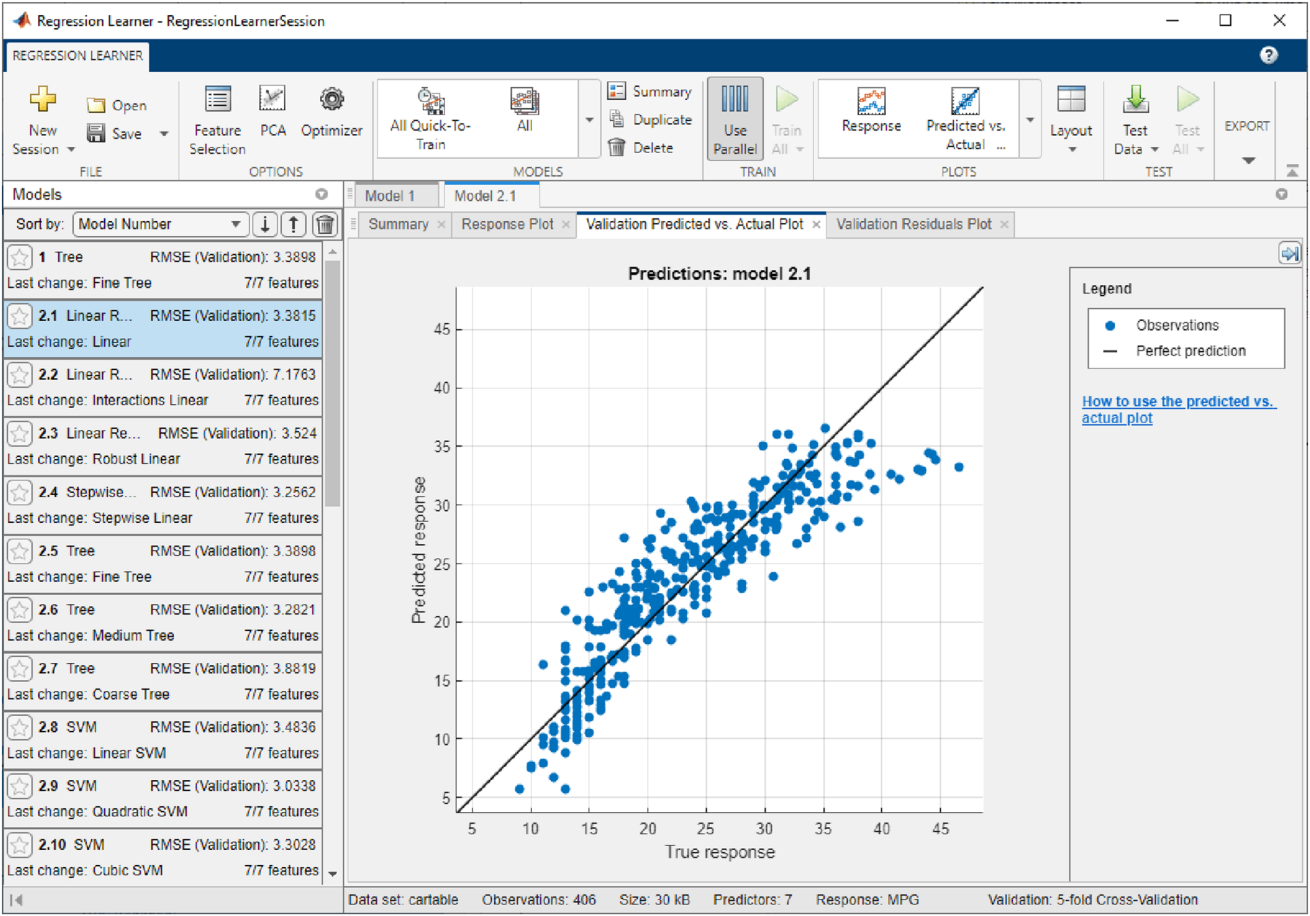
Training results for the selected minimal test error model and correlation graph estimations in the Matlab Regression Learner App [A].

The “Regression Learner App” allows the import of data from columns in a.TXT file and training all or one of the selected methods to form the model applied in testing [E]. It is possible to select some training or testing parameters, e.g. k-fold cross-validation or percentage of data, but all SMLT methods automatically self-optimize model development [A]. After training, a model with minimal testing error is highlighted in a result list to be exported and applied in PQ estimation with unseen data (Fig. 7) [F]. No application interface or additional tool is necessary to obtain Matlab results [G], as the new estimate series of the selected model for the trained output are produced in column format of a defined table by processing unknown.TXT data, using the exported compact model in the main command window.

## PQ-model step by step improvements using additional data input estimations

**Figure 8:**
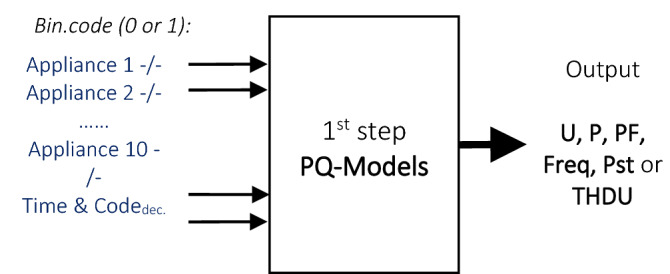
PQ-Modeling using binary coded load inputs in the 1st stage.

The multistage PQ model development uses the initial binary defined codes “0” or “1” of the load combinations of 10 attached household equipment in the first step. PQ data records are not at disposal in this starting phase for the selected appliances of unknown load sequences (Fig. 8). The decimal load code of the connected equipment is applied together with the 10-input binary state and 1 min stamp to determine the harmonics of the signal series.

**Figure 9:**
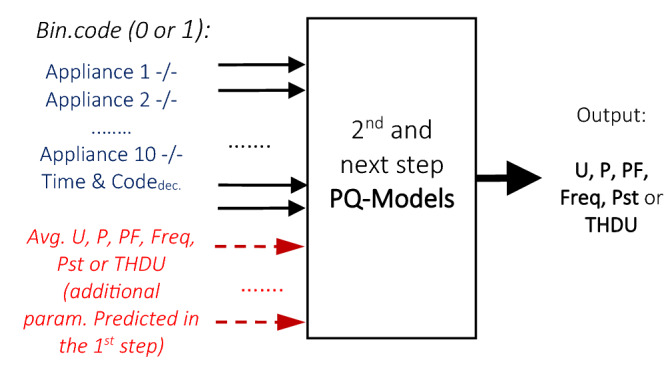
The input of the PQ model vector is extended by processing data of the additional PQ-correlated parameters predicted in the previous stage.

All target PQ outputs of U, THDU P, PF, Pst, and Freq are modelled using 2/3 of the data samples in the training. The resulting final model is tested with the reserved unlearned 1/3 part of the rest data to compute the output PQ series (Fig. 9). These approximate data, predicted by the PQ models in the previous 1st stage, are supplied in the second processing phase for the selected PQ inputs to compute new model estimates.

Measurement of data for correlated PQ parameters can extend the input vector in their next sampled training. After testing, the last predicted PQ approximates, which are missing in the unknown load processing sequences, can be applied to the new developed extension models. Additional PQ inputs allow us to form the next generation models in a more compact and representative structure to get better PQ approximation quality (Fig. 7). The algorithm described above expands step by step the input vector of the next-phase PQ models with applicable parameters. Optimal selection of one or more inputs is inevitable in obtaining an improvement in the accuracy of the model in the next computation.

*Cross-validation of K-folds* is a well-known approach applied in the effective testing of AI models. Random subsets of *K-folds* of the same size are partitioned from the training set. Each part of the testing subset, named fold, is used in the *k*-cross validation of the developed model, opposite to the rest of the *k-1* folds, which means subsets, used in training. This verification strategy is repeated in *k*-cross validation cycles for each particular subset (fold) [C].

## Multistage PQ-pattern modeling and estimation: data experiments

**Figure 10:**
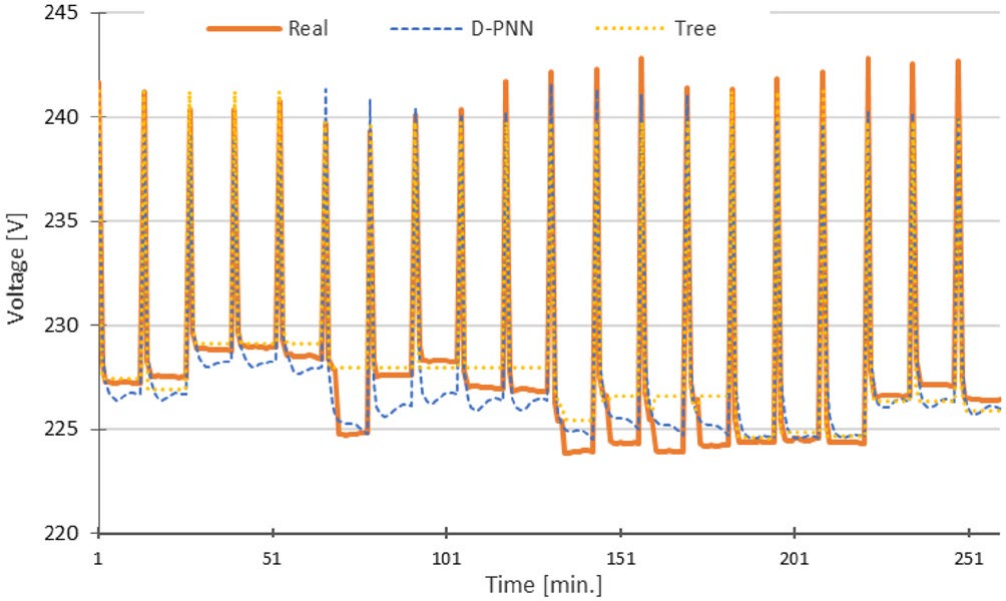
Voltage U—RMSE: D-PNN = 1.153, SMLT (Tree) = 1.118 [V], 12 inputs + THDU (both D-PNN and SMLT) in the 2nd stage.

**Figure 11:**
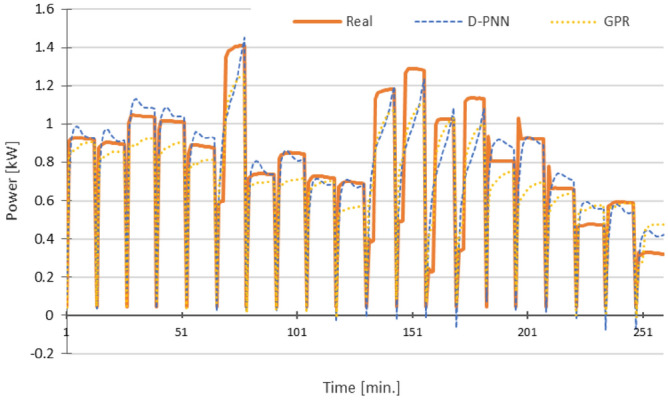
Power P—RMSE: D-PNN = 0.177, SMLT (GPR) = 0.182 [kW], 12 inputs + U + PF (D-PNN), 12 inputs + PF + THDU (SMLT) in the 3rd stage.

**Figure 12:**
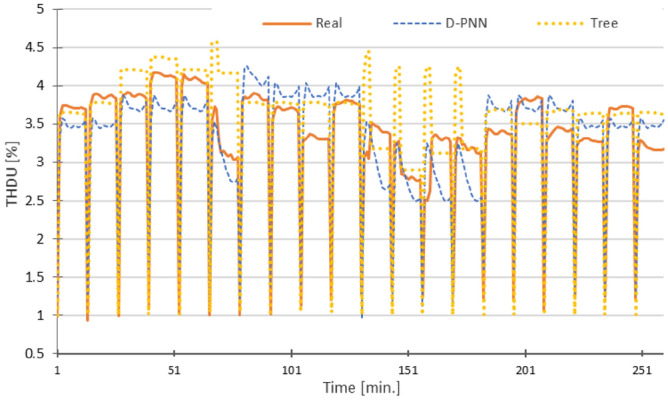
THDU—RMSE: D-PNN = 0.429, SMLT (Tree) = 0.443 [%], 12 inputs + U (both D-PNN and SMLT) in the 2nd stage.

**Figure 13:**
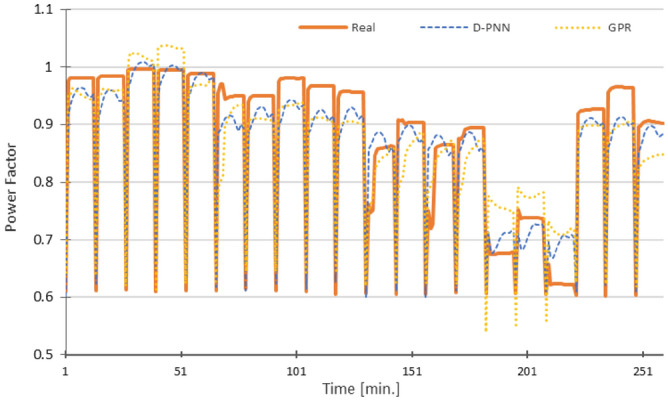
Power Factor PF—RMSE: D-PNN = 0.0516, SMLT (GPR) = 0.0496 [], 12 inputs + Pst + U (D-PNN, 12 inputs + Pst + THDU (SMLT) in the 3rd stage.

**Figure 14:**
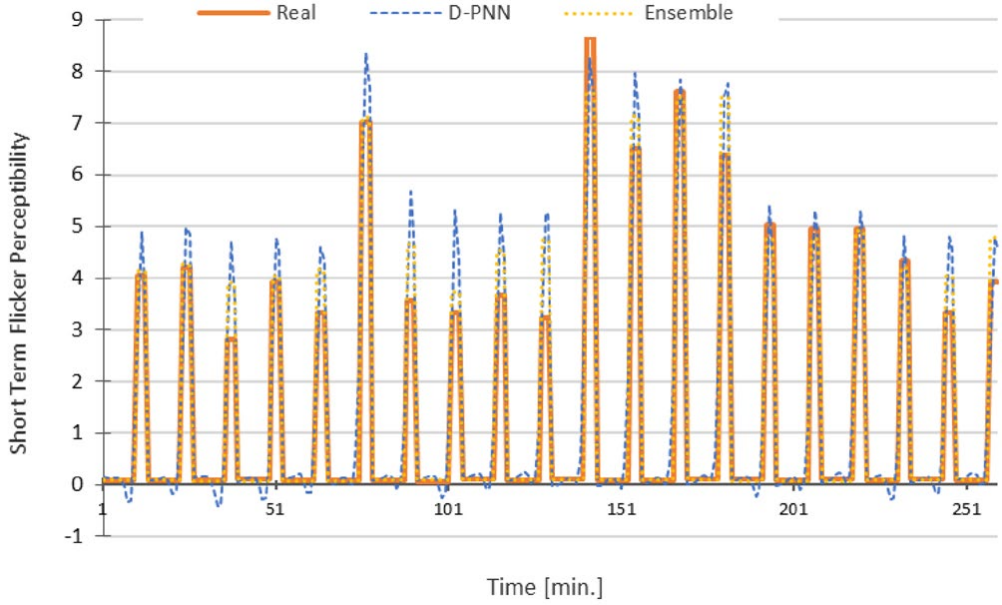
Pst—RMSE: D-PNN = 0.811, SMLT (EBoosT) = 0.544 [], 12 inputs + Pst (both D-PNN and SMLT) in the 2nd stage.

The 2/3 part of the available data were used to develop the models in learning and test, opposite to the 1/3 rest part, used in the PQ estimation (Fig. 8). All the PQ-models, evolved in the 1st phase, have only 10 binary load inputs of the unknown load equipment series at disposal, together with the decimal load code and time stamp, which corresponds to 10 + 2 inputs, to compute the target output. The new estimates for the PQ series correspond to 12 min. measurement range of the particular active 3 electric consumers. That is, 40 combinations, representing 1/3 of the data, were predicted from the total of 120 record samples. The demonstration Figs. 10, 11, 12, 13 and 14 show the 3 modelling extension phases, increasing pattern representation and computability of the models [H].

The graphs in Figs. 10, 11, 12, 13 and 14 demonstrate the particular PQ series of each estimated parameter in 12 min. load sequences only. The 18-min recharging interval in the overall 30-min data cycle was not considered in training, testing, and estimation. The ramping events correspond to the end/initialization of the previous/next measurements, followed by 100% accumulator bank recharging in the complete 30-min. for the evaluated 3-combination load of switched-on household equipment. The graphs display only representative parts of the estimated complete PQ series on the reserved 1/3 data for untrained load sequences to enable a detail resolution scale in Figs. 10, 11, 12, 13 and 14.

Estimates of the D-PNN and SMLT models were obtained by evaluating their test error minima for the untrained reserved part of the data using the described fivefold cross-validation [C]. The most applicable SMLT models were recognized: GPR, EBT, and SVM processing the same input–output data samples as D-PNN (Fig. 8). Their model estimates are the best accurate and vary only in small ranges. Principal component analysis (PCA) is an SMLT input variable extraction option. However, the application of PCA in data preprocessing did not provide any improvement in the accuracy of the PQ models [A]. Optimal input selection was made from the available correlated PQ parameters in the second and third experimental phases of the algorithm to obtain the best improvement in the accuracy of the next-generation PQ models. The selected parameters formed additional vector inputs in the next development and computation process (Fig. 9). The next stage models with the expanded structure were formed for the available data samples to process PQ estimates, calculated in the previous approximation step to improve the quality of new estimates. This progressive refining and re-estimation procedure increases the capability and representability of the models, by step-by-step decreasing the model errors.

## PQ-data processing in multistage computing approximation: evaluation of experimental results

Tree or EBT techniques were recognized as the best SMLT modelling approaches, using only binary input load data and the corresponding decimal code of connected power equipment in the initial primary development level. The evaluated tree-ensemble models of the SMLT methods have the most representative structure for the binary character of input data, applicable in the first starting phase (Fig. 7). Their tree branch lines are chosen step by step to set the response to a leaf node in the binary decision process. The input of the model is extended by additional PQ parameters in the next calculation. The increased complexity in pattern representation yields better results by the use of more sophisticated computational probabilistic principles, such as GPR or EBT, in the next model involvement. GPR models, based on the probability distribution in the definition space, produce the best results in the next stages of approximate data processing [D]. The new added optimal PQ inputs enable one to evolve models with a more detailed adaptable structure and process PQ estimates obtained from the previous computing stages (Tables 1, 2, 3, 4, 5). The applied multilevel adaptation procedure enables us to approximate the target output with progressive quality growth for most of the experimental cases.

Variances in frequency spikes are probably induced by abnormalities in the PQ measurement (Fig. 3). The anomalies have no correlation with the data of other PQ parameters and may result from system oscillations. A small drop in the prediction accuracy of P, PF and Pst using D-PNN models in the next estimation levels might result from an inconvenient selection of PQ inputs or inadequacy in training data patterns. The output of D-PNN models converges better to the real values if more parameters are added to the input vector when processing the previous data estimates in the next approximation phases. D-PNN applies an efficient node input self-detection optimization algorithm in training. However, the variances of the output series are marginal. Polynomial tree networks are generally not able to adequately model binary input data in an acceptable quality. All AI methods, applied in the study, can mostly approximate the target PQ series with increasing accuracy (Figs. 10, 11, 12, 13 and 14) using the real-valued training and supporting estimate data, even in the initialization/end time measurement spikes followed by full recharging cycles.

## Discussion

PQ estimate series are possible to express by time-irregular circular functions. The periods represent time-waves related to the load specifics of the attached power equipment. D-PNN employs the standard GMDH computing approach based on 2-variable processing polynomial functions without an activation, calculated to produce node output and compose ratio- or periodic components, inversely transformed and summed in the PDE model. The sine and cosine Laplace images of sub-PDE derivatives^30^ can be used to model distorted partial-wave functions at the nodes. This periodic PDE conversion, used to represent corrupted PQ cycles, allows one to obtain a better approximation ability than using a separate polynomial-type model. Limitations in computing node output polynomials are partially eliminated by the proposed and published sigmoidal transformation of squared members in itemized equations applied to rationally converted PDE components^27^. The proposed multilevel model extension and development refinement algorithm can be improved in several aspects in future research work.

- Selection of more than one correlated input in the next-stage model development.
- Time-lagged data inputs to use more detailed training series.
- Growth in the complexity of code definition for input binary load combinations.
- Additional or decomposed data used in different computing levels to improve the model convergence.

## Conclusions

The proposed multilevel PQ feature extraction and model extension algorithm was validated using real-world data. Its efficiency was tested and approved for the gradual improvement of AI modelling capability. Model experiments were carried out on real PQ data sets measured in the detached system for all 120 combinations of 3 attached binary-coded load consumers, that is, 10 in all. The main inclusion findings and implications resulting from the data experiments:

- The approximate series of the extra chosen PQ parameters, calculated in the previous data-approaching step, are shared by the next-generation models in the parallel processing to compute better estimates.
- The presented hierarchical AI modelling approach progressively raises the starting level of processed data, expressed initially in binary sequences only, to improve the modelling basis
- The enhanced structure of models is gradually improved along with the quality of approximation on unseen data.
- Real-valued PQ-data samples, used in model development, are extended with PQ approximate series for unknown load combinations in the estimation stage.
- The stepwise evolution strategy enables modeling complex dynamical systems at several categorical levels, where real-valued data parameters are unavailable or insufficient in approximation of the next-time states.
- The advanced data-producing algorithm (Sect. 4) can be applied in modelling of various unseen combinations of load-attached equipment and adapted by most of AI computing techniques (overviewed in Sect. 1) to improve their modelling adaptability for limited or missing input data processed in computing estimates.
- The presented procedure can be additionally improved from the point of optimal input extraction, that is, added one by one or at all, and data processing based on distribution statistics, data segmentation, etc.
- The selection of additional input variables would improve the incremental modelling process using more sophisticated AI techniques, as only a few PQ parameters were evaluated in the experiments.

The most valuable SMLT models were binary trees in the starting 1st level, since the related type of input data was processed by PQ models. The extra inputs enable us to get a better adaptability in the model evolution at the next computation levels by applying more elaborate AI strategies such as D-PNN, GPR, or EBT. (Re)charging of the accumulator backup is to be considered in the system modelling and operational planning of power consumption in addition to RE production depending on weather sources. Extraction and decomposition of the most valuable environmental observation and PQ measurement data into optimal training and testing sets, based on pattern similarity and comparative analysis between historical and forecast data, can help solve some problems in RE power utilization and optimal load scheduling of smart grids.

**Table 1:** Voltage U 3-step model estimations.

Stage	Model inputs		Èrror [V]	
	D-PNN	SMLT	D-PNN	SMLT
1	10 × binary combination load, the decimal code, time (12 inputs)		1.21	1.40
2	+ THDU	+ THDU (Tree)	1.15	1.12
3	+ THDU, Pst	+ THDU, Pst (Tree)	1.10	1.07

**Table 2:** Power P 3-step model estimations.

Stage	Model inputs		Èrror [kW]	
	D-PNN	SMLT	D-PNN	SMLT
1	10 × binary combination load, the decimal code, time (12 inputs)		0.168	0.195
2	+ PF	+ PF (Tree)	0.172	0.193
3	+ PF, U	+ PF, THDU (GPR)	0.170	0.182

**Table 3:** THDU 3-step model estimations.

Stage	Model inputs		Èrror [%]	
	D-PNN	SMLT	D-PNN	SMLT
1	10 × binary combination load, the decimal code, time (12 inputs)		0.408	0.579
2	+ THDU	+ THDU (Tree)	0.430	0.443
3	+ THDU, Pst	+ THDU, Pst (GPR)	0.378	0.393

**Table 4:** Power Factor PF 3-step model estimations.

Stage	Model inputs		Èrror [%]	
	D-PNN	SMLT	D-PNN	SMLT
1	10 × binary combination load, the decimal code, time (12 inputs)		0.0501	0.0439
2	+ THDU	+ THDU (Tree)	0.0507	0.0432
3	+ THDU, Pst	+ THDU, Pst (GPR)	0.0516	0.0496

**Table 5:** Pst 3-step model estimations.

Stage	Model inputs		Èrror []	
	D-PNN	SMLT	D-PNN	SMLT
1	10 × binary combination load, the decimal code, time (12 inputs)		0.797	0.507
2	+ THDU	+ THDU (EBT)	0.810	0.543
3	+ THDU, Pst	+ THDU, Pst (Tree)	0.766	0.546

## Supplementary Information

Supplementary Information.

## Data Availability

All data generated or analyzed during this study are included in this published article [and its supplementary information files]. [A] Matlab—Statistics and Machine Learning tool-box for regression (SMLT) www.mathworks.com/help/stats/choose-regression-model-options.html. https://mathworks.com/help/stats/select-data-and-validation-for-regression-problem.html. [B] Matlab—Support Vector Machine (SVM) regression. www.mathworks.com/help/stats/understanding-support-vector-machine-regression.html. [C] Matlab—k-fold cross validation www.mathworks.com/discovery/cross-validation.html. [D] Matlab—Gaussian Process Regression (GPR) www.mathworks.com/help/stats/gaussian-process-regression-models.html. [E] Matlab—Regression Learner App www.mathworks.com/help/stats/regression-learner-app.html?s_tid=CRUX_lftnav. [F] Train Regression Models in Regression Learner App www.mathworks.com/help/stats/train-regression-models-in-regression-learner-app.html. [G] Export Regression Model to Predict New Data www.mathworks.com/help/stats/export-regression-model-to-predict-new-data.html. [G] Applied PQ-data sets (in Excel.xls) https://drive.google.com/drive/folders/1ZAw8KcvDEDM-i7ifVe_hDoS35nI64-Fh.
